# Mild hypothermia does not attenuate platelet aggregation and may even increase ADP-stimulated platelet aggregation after clopidogrel treatment

**DOI:** 10.1186/1477-9560-7-2

**Published:** 2009-02-23

**Authors:** Carl Högberg, David Erlinge, Oscar Ö Braun

**Affiliations:** 1 Department of Cardiology, Lund University, Lund, Sweden

## Abstract

**Background:**

Mild hypothermia is currently standard of care for cardiac arrest patients in many hospitals and a common belief is that hypothermia attenuates platelet aggregation. We wanted to examine the effects of clopidogrel on platelet aggregation during hypothermia.

**Methods:**

Platelet reactivity at 37°C and 33°C was evaluated by light transmission aggregometry and vasodilator-stimulated phosphoprotein (VASP) in blood from healthy volunteers before, and 24 hours after, a 600 mg loading dose of clopidogrel.

**Results:**

Collagen, 5-HT, epinephrine, U46619 and ADP-induced platelet aggregation was unaltered or even increased by hypothermia. After clopidogrel, there was a significant increase in platelet aggregation for 5 and 20 μM ADP at 33°C compared to 37°C (46 ± 5 vs. 34 ± 5% and 58 ± 4 vs. 47 ± 4%, p < 0.001, n = 8). Hypothermia also increased ADP-induced aggregation after pretreatment with the P2Y_1 _antagonist MRS2500. The decreased responsiveness to clopidogrel during hypothermia could be overcome by addition of the reversible P2Y_12 _antagonist AZD6140. ADP-induced inhibition of VASP-phosphorylation was unaffected by hypothermia both in the presence and absence of clopidogrel. A dose-response curve for ADP-induced platelet aggregation revealed increased potency for ADP during hypothermia with no difference in efficacy.

**Conclusion:**

Mild hypothermia did not attenuate platelet aggregation, instead it even increased ADP-stimulated platelet aggregation after clopidogrel treatment. Dual platelet inhibition with aspirin and a P2Y_12 _receptor antagonist is probably needed for patients with acute coronary syndromes treated with mild hypothermia, and it is possible that future ADP blockers could be of benefit.

## Introduction

Hypothermia is a condition in which many biological reactions are altered. Even a minor change of temperature in a cell can alter the response to stimuli. Mild hypothermia (33–35°C) has been shown to reduce mortality and improve neurological outcome in unconscious patients with cardiac arrest [1,2], and is recommended by treatment guidelines [3]. Mild hypothermia is already standard of care in many hospitals for cardiac arrest patients. Furthermore, mild hypothermia has been shown to reduce myocardial infarct size in animal models, and clinical studies are ongoing to determine whether this strategy can preserve not only the brain but also the heart [4]. For many patients with cardiac arrest, the aetiology is an acute coronary syndrome, which needs to be treated with platelet inhibitors. The cornerstone of this treatment is dual antiplatelet therapy with aspirin and clopidogrel [3]. However, to our knowledge, the pharmacodynamic effects of clopidogrel during hypothermia have never been studied.

Conventional wisdom holds that hypothermia reduces platelet activation, and it has been suggested that antiplatelet treatment should be reduced during hypothermia. There are some studies supporting this notion [5-7]. However, there are more recent reports suggesting increased platelet reactivity during mild hypothermia [8-11]. It is of great importance to select the correct level of platelet inhibition for this patient category. Insufficient platelet inhibition may result in stent thrombosis, acute myocardial infarction, and cardiac death. It is therefore important to understand the effect of hypothermia on clopidogrel treatment.

To examine the effect of clopidogrel during mild hypothermia, we analyzed platelet reactivity as measured by light transmission aggregometry (LTA), vasodilator-stimulated phosphoprotein (VASP)-phosphorylation, and a P2Y_12 _specific flow cytometry assay, in healthy volunteers at 37° and 33°C before and 24 hours after a 600 mg dose of clopidogrel.

## Materials and methods

### Platelet preparation for LTA analysis

Whole blood samples (40 mL) were collected from healthy voluntary blood donors (n = 8). The blood samples were collected from an antecubital vein into Becton Dickinson (BD) Vacutainer™ tubes containing 0.129 M sodium citrate. A second blood sample was collected 24 hours after a 600 mg oral loading dose of clopidogrel. To obtain platelet rich plasma (PRP), whole blood was centrifuged (10 min, 140 × g, room temperature (RT)). After centrifugation the supernatant containing the PRP was collected and transferred to a 15 mL polypropylene tube. To obtain reference platelet poor plasma (PPP), whole blood was centrifuged (10 min, 2260 × g, RT). The supernatant containing PPP was collected into a 15 mL polypropylene tube.

### LTA analyses

Two serial connected aggregometers (Chrono-Log 490, Chrono-Log Corporation, Havertown, PA, USA) were preheated to 37°C and 33°C respectively. To allow for temperature equilibration, the PRP was incubated for 5 minutes in the aggregometers prior to addition of an agonist. In experiments with ex vivo antagonists, addition of these compounds was followed by incubation for 5 min prior to ADP stimulation. Analyses were performed using AGGRO/LINK^® ^software (Chrono-Log Corporation, Havertown, PA, USA.). Stirring was set to 1200 rpm and the optical band was in the range of 600 OD. Each of the experiments continued for 6 minutes (to observe if a secondary aggregation took place). The test volumes containing PRP were set to 250 μL, while the PPP volume in the reference wells of the two aggregometers was set to 500 μL.

### Flow cytometric analysis of VASP-phosphorylation

Whole blood was collected from the antecubital vein into BD Vacutainer™ tubes containing 0.129 M sodium citrate. The blood was incubated for 10 min at 33°C and 37°C. The VASP assay was performed using the platelet VASP/P2Y_12 _kit (Biocytex Platelet VASP kit, Marseille, FR). The experimental procedure was carried out according to the manufacturer's instructions except that the samples were prepared at 33°C and 37°C instead of room temperature until the fixation step. Mean Fluorescence Intensity (MFI) was measured with a flow cytometer (FACScalibur^®^, BD, USA). Platelet reactivity index (PRI) was calculated from the corrected MFI (cMFI) of prostaglandin E_1 _(PGE_1_) and ADP- and PGE_1_- treated samples according to the following equation:

PRI = [(cMFI_(PGE1) _- cMFI_(PGE1+ADP)_)/cMFI_(PGE1)_] × 100

### Drugs

ADP, epinephrine, collagen, and thrombin were from Chrono-Log Corporation, USA. AZD6140 was a gift from Astra-Zeneca, Sweden. MRS2500 was from Tocris Bioscience, UK. U-46619 was from Sigma-Aldrich, USA. Clopidogrel was from Sanofi Pharma Bristol-Myers Squibb SNC, France. All drugs (clopidogrel excluded) were dissolved in 0.9% saline; AZD6140 was dissolved in DMSO at 10^-^2 M and then further diluted in 0.9% saline.

### Ethics

The Ethics Committee of Lund University approved the project. All blood donors provided signed informed consent to participate in the study.

### Calculation and statistics

Statistical analyses were performed using the GraphPad Prism 4.0 software (Graph Pad Software, USA). LTA and VASP data were analyzed using paired Student's t-test. P-values less than 0.05 were regarded as statistically significant. Values are presented as mean ± s.e.m.

## Results

### ADP-stimulated platelet aggregation evaluated by LTA

**Figure 1:**
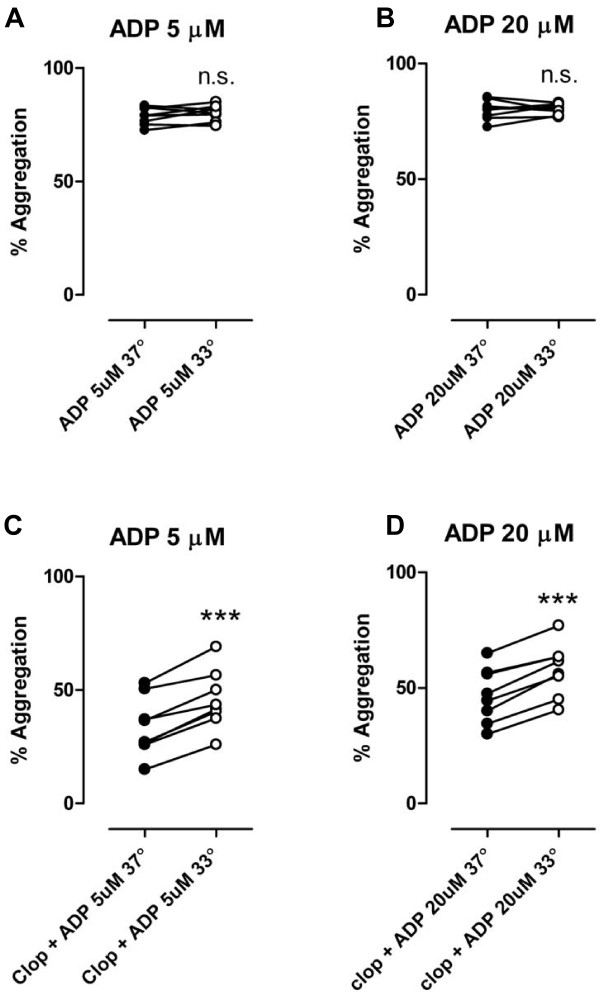
**ADP-stimulated platelet aggregation at 5 and 20 μM [a, b] and after treatment with clopidogrel (600 mg) po [c, d]**. n = 8. filled symbols represents normothermia (37°), open symbols represents hypothermia (33°), n.s. = not significant, *** = P < 0.001.

There was no significant difference in maximum platelet aggregation (MPA) levels when PRP was stimulated with 5 μM ADP at 33°C compared to 37°C (79 ± 1 vs. 80 ± 1%, P = NS, n = 8) or with 20 μM ADP at 33°C compared to 37°C (80 ± 2 vs. 80 ± 1%, P = NS, n = 8) [Figure 1a, b].

### ADP-stimulated platelet aggregation before and after clopidogrel treatment

**Figure 2:**
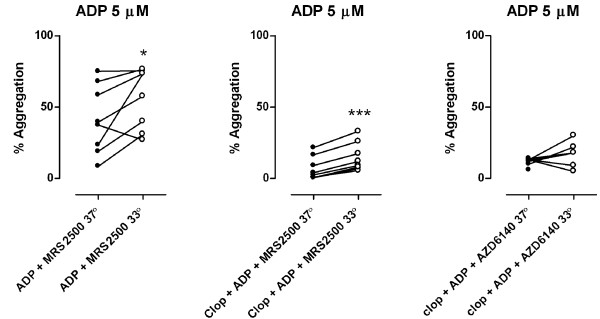
**ADP (5 μM) -stimulated platelet aggregation after pretreatment with MRS2500 (1 μM) *ex vivo *[a], after pretreatment with clopidogrel (600 mg) *po *and MRS2500 *ex vivo *[b], and after pretreatment with clopidogrel (600 mg) *po *and AZD6140 (10 μM) *ex vivo *[c]**. n = 8. Filled symbols represents normothermia (37°), open symbols represents hypothermia (33°), n.s. = not significant, * = P < 0.05, *** = P < 0.001.

24 hours after a loading dose of 600 mg clopidogrel, there was a significant increase in MPA after both 5 and 20 μM ADP at 33°C compared to 37°C (46 ± 5 vs. 34 ± 5%, P < 0.001, n = 8, and 58 ± 4 vs. 47 ± 4%, P < 0.001, n = 8) [Figure 1c, d]. There was a significant increase in MPA when PRP treated with the P2Y_1 _receptor antagonist MRS2500 (1 μM) was stimulated with 5 μM ADP at 33°C compared to 37°C (57 ± 7 vs. 41 ± 9%, P < 0.05, n = 8) [Figure 2a] and with combined treatment with oral clopidogrel and MRS2500 *ex vivo *(15 ± 4 vs. 7 ± 3%, P < 0.001, n = 8) [Figure 2b]. 10 μM AZD6140 *ex vivo *in addition to per oral clopidogrelmarkedly reduced MPA after 5 μM ADP with no significant difference in MPA at 33°C compared to 37°C [Figure 2c].

### Collagen-, 5-HT-, epinephrine-, and U46619-stimulated platelet aggregation before and after clopidogrel treatment

**Figure 3:**
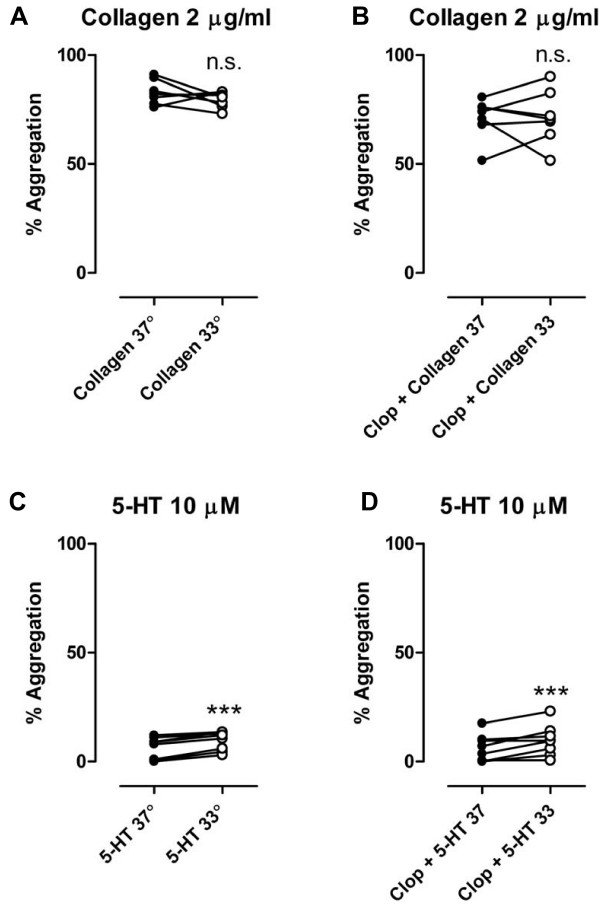
**Platelet aggregation stimulated by collagen (2 μg/ml) [a, b] and by 5-HT (10 μM) [c, d] before and after pretreatement with clopidogrel (600 mg) *po***. n = 8. Filled symbols represents normothermia (37°), open symbols represents hypothermia (33°), n.s. = not significant, *** = p < 0.001.

**Figure 4:**
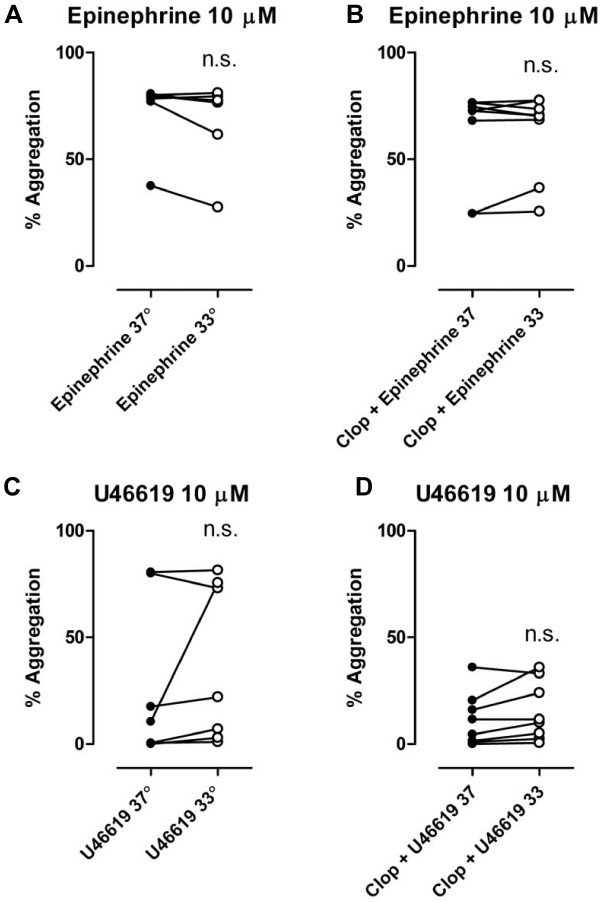
**Platelet aggregation stimulated by epinephrine (10 μM) [a, b] and U46619 (10 μM) [c, d] before and after pretreatement with clopidogrel (600 mg) *po***. N = 6–8. Filled symbols represents normothermia (37°), open symbols represents hypothermia (33°), n.s. = not significant.

There was no significant difference in MPA when PRP was stimulated with 2 μg/ml collagen without clopidogrel at 33°C compared to 37°C (79 ± 1 vs. 83 ± 2%, P = NS, n = 8) or after oral clopidogrel (71 ± 5 vs 71 ± 4%, P = NS, n = 8) [Figure 3a, b]. There was a significant increase in MPA levels with 10 μM 5-HT at 33°C compared to 37°C both without clopidogrel (9.5 ± 1.5 vs. 6.3 ± 1.8%, P < 0.001, n = 8) and after oral clopidogrel (9.6 ± 2.4 vs. 6.0 ± 2.2%, P < 0.001, n = 8) [Figure 3c, d]. No differences in MPA between temperatures were observed with epinephrine 10 μM without clopidogrel (73 ± 6 vs 69 ± 7%, P = NS, n = 8) or after clopidogrel (61 ± 8 vs. 62 ± 7%, P = NS., n = 8) [Figure 4a, b] or with U46619 10 μM without clopidogrel (27 ± 14 vs 38 ± 14%, P = NS, n = 8) or after clopidogrel (15 ± 5 vs. 11 ± 4%, P = NS, n = 8) [Figure 4c, d].

### Inhibition of P2Y_12 _receptors evaluated by VASP-phosphorylation

**Figure 5:**
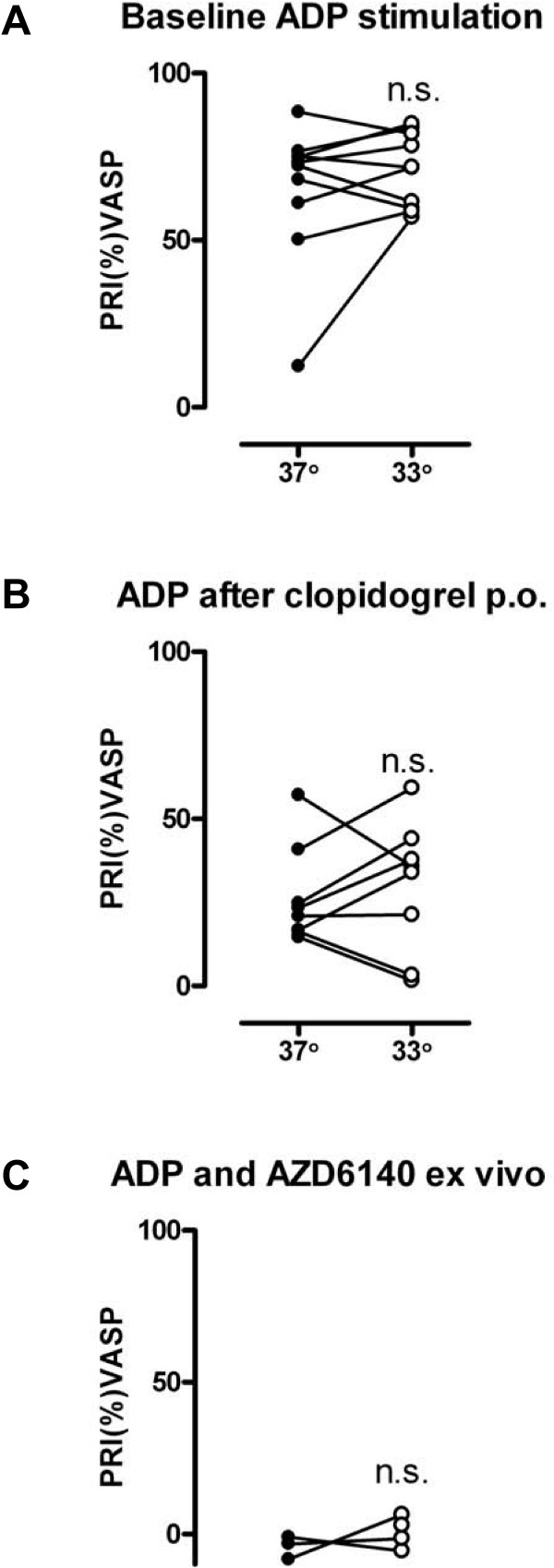
**Flow cytometry study in which phosphorylation of VASP (and hence inhibition of platelet activation) is stimulated by PGE_1 _and dephosphorylation (and hence augmentation of platelet activation) is mediated by ADP**. Platelet activation is represented as platelet reactivity index (%PRI). ADP-stimulated platelet aggregation at baseline **[a]**, after pretreatment with clopidogrel *po ***[b]**, and after pretreatment with AZD6140 (10 μM) *ex vivo ***[c]**. n = 8. Filled symbols represents normothermia (37°), open symbols represents hypothermia (33°), n.s. = not significant.

PRI (%) as assessed by the VASP kit was similar at 33°C compared to 37°C at baseline (71 ± 3 vs. 65 ± 7%, P = NS, n = 8) and after a 600 mg loading dose of clopidogrel (30 ± 7 vs. 27 ± 5%, P = NS, n = 8) [Figure 5a, b]. PRI (%) VASP was also similar at 33°C compared to 37°C with ADP and 10 μM AZD6140 added *ex vivo *[Figure 5c].

### Dose response for ADP-stimulated platelet aggregation evaluated by LTA

**Figure 6:**
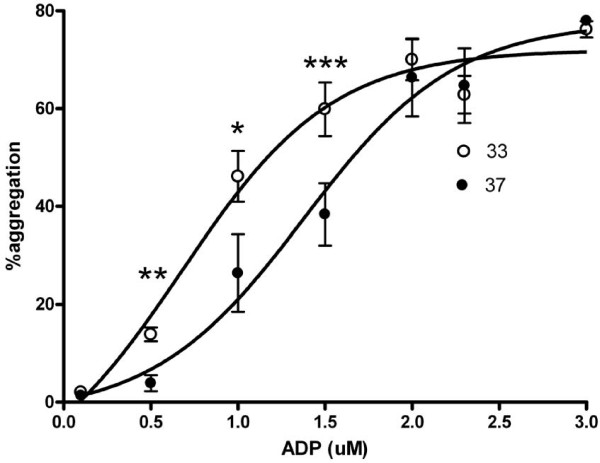
**ADP dose response curve for platelet aggregation at normothermia and hypothermia**. Filled symbols represents normothermia (37°), open symbols represents hypothermia (33°), * = p < 0.05, ** = p < 0.005, *** = p < 0.001.

To further evaluate the mechanism of increased sensitivity to ADP during hypothermia, we performed a narrow dose response curve for ADP-induced platelet aggregation in a new subset of healthy individuals. There was no significant increase in the plateau phase of MPA at 33°C compared to 37°C, 72.0% (95% confidence interval (CI) 66.9–77.2) vs 77.8%, CI 66.9–88.6), P = NS, n = 7) [Figure 6]. However, there was leftward shift of the dose response curve indicating increased potency for ADP during hypothermia (EC_50 _= 0.68 μM (confidence interval 0.41–0.93)) compared to normothermia (EC_50 _= 1.38 μM (confidence interval 1.06–1.70)). MPA was significantly higher during hypothermia for 0.5, 1.0, and 1.5 μM of ADP.

## Discussion

We examined platelet reactivity before and after clopidogrel treatment at 37°C and at 33°C, a temperature used for the treatment of patients resuscitated after cardiac arrest. Mild hypothermia did not attenuate platelet aggregation. Instead, collagen, 5-HT, epinephrine, the thromboxane analogue U46619 and ADP-induced platelet aggregation was unaltered or even increased by hypothermia. Dose-response curves revealed increased potency of ADP during hypothermia with no change in efficacy (maximum effect) compared with normothermia. Furthermore, the inhibitory effect of clopidogrel was attenuated. Similar attenuation of effect on ADP-induced aggregation was also seen after inhibition with the P2Y_1 _antagonist MRS2500. The decreased responsiveness to clopidogrel during hypothermia could be overcome by addition of the P2Y_12 _antagonist AZD6140.

Dual antiplatelet therapy with aspirin and clopidogrel is recommended treatment for patients with acute coronary syndromes and after stent implantation [3]. A more rapid onset and higher level of platelet inhibition can be obtained with a 600 mg loading dose of clopidogrel, a dose recently endorsed for acute coronary syndromes to obtain better protection during percutaneous coronary interventions. A large number of patients with cardiac arrest have an acute coronary syndrome; some of them are treated with direct-PCI and stenting because of ST-elevation myocardial infarction, and therefore receive clopidogrel treatment. Despite this, the effect of clopidogrel has to our knowledge never been examined during mild hypothermia.

There is a general belief that mild hypothermia attenuates platelet aggregation [6,12], and it was somewhat surprising to find that collagen, 5-HT, epinephrine, the thromboxane analogue U46619 and ADP-stimulated platelet aggregation was unaltered or even increased by mild hypothermia. Most of the previous studies have been performed at lower temperatures, eg, 28°, 22°, or even 2°C [6,7,13]. Our interest was in the growing use of mild hypothermia (33–35°C) for the treatment of cardiac arrest, and perhaps in the future to reduce infarct size in patients with acute myocardial infarction. One study demonstrated a reduction in ADP-stimulated platelet aggregation by 16% at 32°C compared to 37°C [5]. In contrast, a recent study by Heptinstall et al. demonstrated *increased *ADP-stimulated platelet aggregation at 28°C [9]. Lindenblatt et al[8] found increased *ex vivo *stimulated GpIIb/IIIa expression after TRAP stimulation at 34°C compared to normothermia, as well as accelerated thrombus formation in vivo in mice at 34°C. Our findings are also in agreement with Scharbert and co-workers[10], who found that platelet aggregation was increased with ADP, but unaltered with collagen during hypothermia. Further, shear stress-induced aggregation has been shown to increase at 32°C and 35°C[11].

In the present study, we found an attenuated effect of clopidogrel on ADP-stimulated platelet aggregation during mild hypothermia. The effect was highly significant and consistent at both doses of ADP used. This is in agreement with previous studies that have shown increased ADP responses during hypothermia in the presence of aspirin[9,10]. However, mild hypothermia has also been shown to augment the inhibitory effect of the reversible GpIIb/IIIa-blockers eptifibatide and tirofiban (but not that of the irreversible blocker abciximab)[5]. It could be hypothesized that hypothermia affects different classes of platelet inhibitors differently. Such information is of course important in the selection of adequate platelet inhibition for patients treated with mild hypothermia.

Since the first report of a variable response to clopidogrel [14], a large number of studies have found a high prevalence of patients with pharmacodynamic poor responsiveness to clopidogrel [15]. Our data indicate that hypothermia induces a situation in which the effect of clopidogrel is reduced. The decreased responsiveness to clopidogrel during hypothermia could be overcome by addition of the reversible P2Y_12 _antagonist AZD6140.

ADP activates two receptors on the platelet: the Gi-coupled P2Y_12 _receptor coupled to inhibition of cAMP; and the Gq-coupled P2Y_1 _receptor, which stimulates IP_3 _and increases intracellular Ca^2+^. We tried to elucidate the mechanisms of the platelet effects induced by hypothermia by testing whether they were P2Y_12_-specific. We added the selective P2Y_1_-receptor antagonist MRS2500. Even in this situation, increased ADP sensitivity was seen during mild hypothermia. The effect was even more pronounced when MRS2500 was combined with clopidogrel. Thus, the increased platelet stimulatory effect of ADP can be seen regardless of which ADP receptor is blocked.

ADP activation of the P2Y_12 _receptor results in reduction of cAMP. VASP is an intracellular actin regulatory protein [16,17] that is phosphorylated into its P-VASP form by cAMP dependent protein kinases. ADP, the natural agonist to the P2Y_12 _receptor, inhibits VASP phosphorylation through inhibition of adenylate cyclase and downregulation of cAMP production. We examined if the mechanism of the platelet effects of hypothermia are mediated at the level of VASP-phosphorylation. However, we saw no effect of hypothermia on VASP-phosphorylation when whole blood was stimulated by ADP alone or in combination with clopidogrel or AZD6140.

The mechanism of the increased ADP-induced platelet aggregation during hypothermia observed in our study remains elusive. It does not seem to depend on antagonist-receptor interaction or intracellular second messenger systems. Another possible explanation would be altered degradation rates of ADP. However, we repeated the experiments with the stable ADP analogue 2-MeSADP and obtained similar results (data not shown). To understand the receptor pharmacology during hypothermia, we assessed dose-response curves for ADP. Platelets at 33°C displayed an increased sensitivity to ADP and epinephrine compared to 37°C, with a leftward shift of the dose-response curve and a significantly lower EC_50 _value for platelets at 33°C. Thus, it was only possible to see a difference in effect when doses in the sigmoidal part of the curve were tested, with the maximum effect being unaltered compared with normothermia. This observation may explain some of the conflicting data in the literature. It also indicates that it is important to take the concentration of the agonist into consideration when studying hypothermia and platelets.

Haemostasis during hypothermia is complex, and it is possible that platelet responses differ at different temperatures. We found unaltered or even increased platelet responses during mild hypothermia. The literature contains conflicting data, but in support of our findings, increased platelet activation has been seen during profound hypothermia[13] and increased aggregation has been reported at a more intermediate temperature of 28°C [9]. However, prolongation of bleeding time has been reported in clinical situations with hypothermia [18-20]. It is possible that this primarily reflects effects of hypothermia on the coagulation system, especially a decrease in the fibrinolysis-inhibiting α_2_-macroglobulin levels [21]. On the other hand, unintentional perioperative hypothermia is associated with postoperative myocardial ischemia, indicating a prothrombotic effect of hypothermia, and platelet count and platelet activity have been shown to be increased in this setting [12,21].

## Conclusion

In conclusion, our study indicates that platelet reactivity is unaltered and in some situations increased during mild hypothermia. The inhibitory effect of clopidogrel was attenuated in our study. The clinical conclusion is that we cannot rely on hypothermia per se as a platelet inhibitor. Based on current *ex vivo *evidence, dual platelet inhibition with aspirin and clopidogrel is probably needed for patients with acute coronary syndromes treated with mild hypothermia. Since hypothermia induces a state of reduced clopidogrel responsiveness, it is possible that new reversible ADP blockers such as AZD6140 could be beneficial. Clinical studies are needed to determine the best use of platelet and coagulation inhibitors for the growing number of patients treated with mild hypothermia.

## Competing interests

The authors declare that they have no competing interests.

## Authors' contributions

CH carried out the platelet function studies, analyzed and participated in writing the manuscript. DE and OB designed the study, analyzed data and wrote the manuscript. All authors read and approved the final manuscript.
